# Bibliometric insight into neoadjuvant immunotherapy in non-small cell lung cancer: trends, collaborations, and future avenues

**DOI:** 10.3389/fimmu.2025.1533651

**Published:** 2025-02-10

**Authors:** Pengliang Xu, Huanming Yu, Hupo Bian, Dan Jia, Wenhui Li, Hongfeng Dong, Xiuhua Peng

**Affiliations:** ^1^ Department of Thoracic Surgery, The First People’s Hospital of Huzhou, Huzhou, China; ^2^ Department of Radiology, The First People’s Hospital of Huzhou, Huzhou, China; ^3^ Department of Respiratory Medicine, The First People’s Hospital of Huzhou, Huzhou, China

**Keywords:** non-small cell lung cancer, neoadjuvant immunotherapy, chemotherapy, CiteSpace, visual analysis

## Abstract

**Background:**

Neoadjuvant immunotherapy (NIT) is a rapidly emerging paradigm for advanced resectable non-small cell lung cancer (NSCLC). However, there is no bibliometric analysis in this research field.

**Objective:**

To analyze the hotspots and trends in the research of NIT for NSCLC and provide a reference for the study of NIT for lung cancer in China.

**Methods:**

Retrieve literature related to NIT for NSCLC from Web of Science, PubMed, and Scopus databases up to September 10, 2024. Use CiteSpace and VOSviewer software visualization software to analyze the keywords of country, author, institution, and literature.

**Results:**

There were 1575 references, and the overall annual publication volume showed an upward trend; Forde and Patrick M have published the most articles in the literature. The research hotspots mainly focus on chemotherapy, NIT for NSCLC, immunotherapy, neoadjuvant chemotherapy, pathological reactions, etc.

**Conclusions:**

This is the first bibliometric study comprehensively summarizing NIT’s research trends and development in NSCLC. Our bibliometric assessment provides a panoramic view of the research milieu surrounding NIT for NSCLC, encapsulating the present state, evolving trends, and potential future directions, particularly emphasizing the promise of immunochemotherapy.

## Introduction

The management of non-small cell lung cancer (NSCLC), particularly locally advanced nonmetastatic cases, continues to evolve. Resectable NSCLC is generally defined as stage I–IIIA disease, utilizing the American Joint Committee on Cancer (AJCC) 8th edition guidelines. In stage I - IIIA non-small cell lung cancer (NSCLC), the tumors are relatively localized without extensive distant metastases, and it is highly important to carry out neoadjuvant therapy. Neoadjuvant therapy can reduce the tumor volume, downstage the disease, increase the rate of complete surgical resection, and improve patients’ long-term survival expectations. Retrospective studies (1) have revealed that the 5-y survival rate of patients at this stage receiving neoadjuvant therapy is significantly improved compared with that of those undergoing surgery alone. Previously, neoadjuvant chemotherapy was regarded as the main approach to improve localized NSCLC. However, with the continuous increase in the number of clinical trials, the benefits of neoadjuvant chemotherapy have been found to be somewhat limited (2). Therefore, there is an urgent need for new neoadjuvant treatment modalities.

Immune checkpoint inhibitors (ICIs) can block the inhibitory signal transduction pathways that suppress the activity of T lymphocytes, thereby increasing the body’s antitumor activity (3). Numerous clinical trials have confirmed that monoclonal antibodies targeting programmed cell death protein 1/programmed death ligand 1 (PD-1/PD-L1) and cytotoxic T lymphocyte-associated protein 4 (CTLA-4) can improve overall survival in patients with advanced non-small cell lung cancer (NSCLC) (4). Multiple clinical trials (5–7) have demonstrated that neoadjuvant immunotherapy (NIT), whether used alone or in combination with chemotherapy, can benefit patients with stage I - IIIA NSCLC in terms of survival.

From a biological perspective, the body’s immune system is relatively intact before surgery, and immunotherapy can fully mobilize various immune cells to participate in the antitumor immune response. Although early-stage tumors may seem localized, there may actually be occult micrometastases that are difficult to reach by traditional surgery. Neoadjuvant immunotherapy acts on the primary tumor before surgery, prompting it to release antigens and activate immune effector cells. These cells can attack the primary tumor and eliminate micrometastases as well (8, 9). Preclinical animal model studies have confirmed this effect (10). Given the rapid development and increasing importance of NIT in clinical applications, a bibliometric review to analyzing in detail the research trends regarding NIT on a global scale is necessary but has not yet been conducted.

The core advantage of visualization mapping software lies in its ability to present massive amounts of literature data in a knowledge field in an intuitive manner through multidimensional, phased, and dynamic analysis techniques. Through intelligent layout design, this tool can automatically identify and highlight the development history and research hotspots in the field. CiteSpace (11) is an open-source software tool used for bibliometric and visualization analysis, aiming to assist researchers in exploring the relationships within information from academic literature, research trends, and collaboration networks. Therefore, this study utilized CiteSpace and VOSviewer software to comprehensively analyze the current status, hotspots, and frontier trends of NIT research on NSCLC both at home and abroad, aiming to provide references for future research and applications in China. This study conducted a thorough analysis of English-language literature in the field of “lung cancer NIT” from the Web of Science, PubMed, and Scopus databases. CiteSpace information visualization software was employed to analyze the trends of published papers, distribution of countries, author cooperation networks, institutional distribution, keyword co-occurrence, cluster analysis, and timeline analysis.

## Materials and methods

### Data sources and retrieval strategies

**Figure 1:**
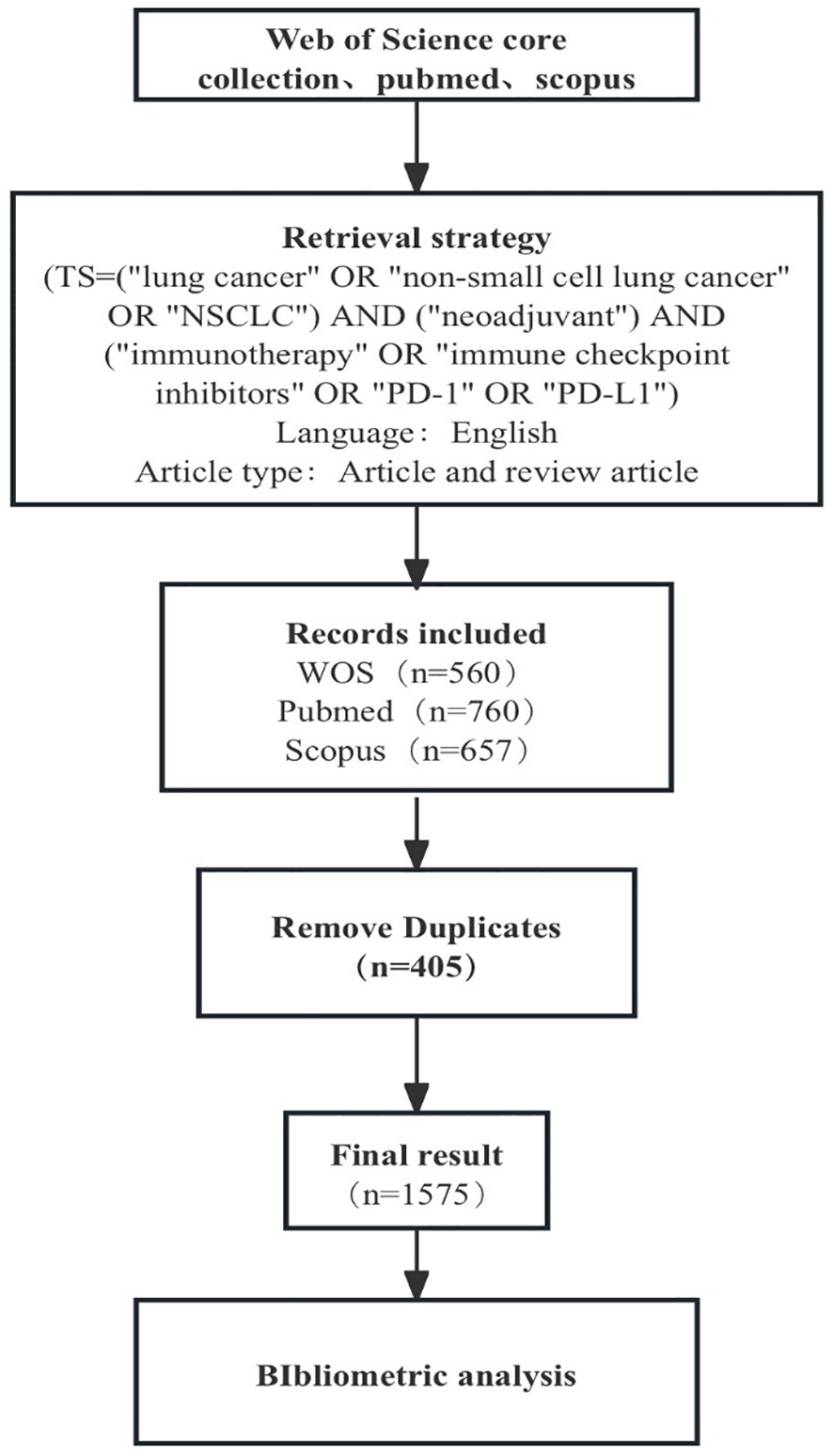
Flowchart of the included articles.

The literature sources used in this study were the Web of Science, PubMed, and Scopus databases. The retrieval criteria were as follows: (TS= “nonsmall cell lung cancer” OR “NSCLC” OR “lung cancer”) AND (“neoadjuvant”) AND (“immunotherapy” OR “immune checkpoint inhibitors” OR “PD-1” OR “PD-L1”); the retrieval time ranged from database establishment to September 10, 2024. A total of 1977 articles were retrieved. We chose an article for analysis and selected “English” as the language. After deduplication, we ultimately included 1575 articles (**Figure 1**).

### Data processing

CiteSpace is an information visualization software that runs in the Java environment. It was developed by Professor Chaomei Chen from Drexel University in the United States and uses path-finding network algorithms to calculate sample literature in a particular field. CiteSpace explores the potential value of the discipline’s transformation and the cutting-edge trends of its future development by visualizing the graphs drawn. This study used CiteSpace scientific literature software to visualize and analyze literature in the NIT for lung cancer from 1991 to 2024, providing a reference for promoting related research on “neoadjuvant immunotherapy for lung cancer.” Articles from the Web of Science, PubMed, and Scopus databases were exported in plain text, Citation Manager, and RIS formats, respectively, and uniformly imported into CiteSpace 6.2.R4 under the name “download” for format conversion. Time partitioning (time slicing=1) selection: 1991-2024; time nodes (years per slice): 1 y. The term sources for the theme were theme, abstract, and keywords; threshold (top N per slice) =10.

## Results

### Trend analysis of publications

**Figure 2:**
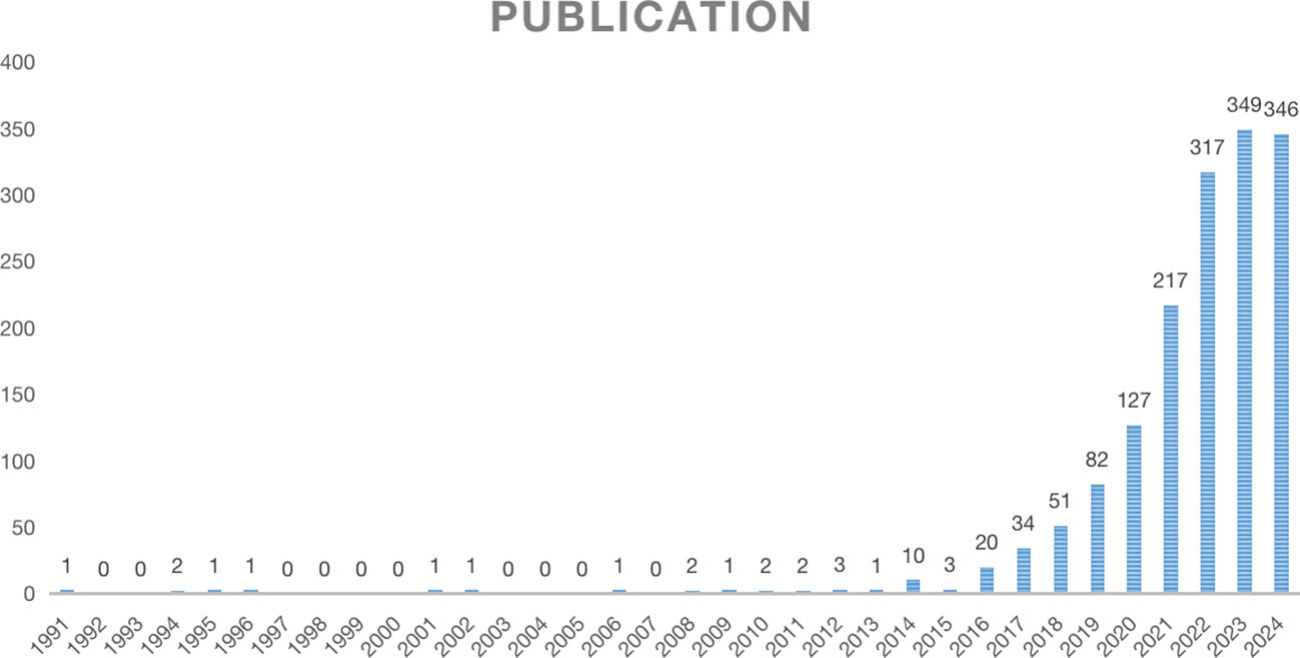
Trend chart of annual publication volume.

An analysis of the annual distribution of 1575 selected studies can, to some extent, reveal the speed, depth, and maturity of the development of the discipline. The yearly publication volume of the literature is shown in **Figure 2**. From 1991 to 2013, the overall development of the number of publications was relatively flat, with low publication volumes of 3 or less. After 2014, the number of published studies in foreign countries rapidly increased, indicating that the field of new immunotherapy for lung cancer has received widespread attention from foreign scholars in the past decade. In 2023, the total number of publications peaked (349 articles). It is expected to achieve new breakthroughs in 2024, indicating increasing attention to the field of NIT for lung cancer. The overall trend continues to rise, highlighting the critical position of NIT for lung cancer research abroad.

### National co-occurrence analysis

**Figure 3:**
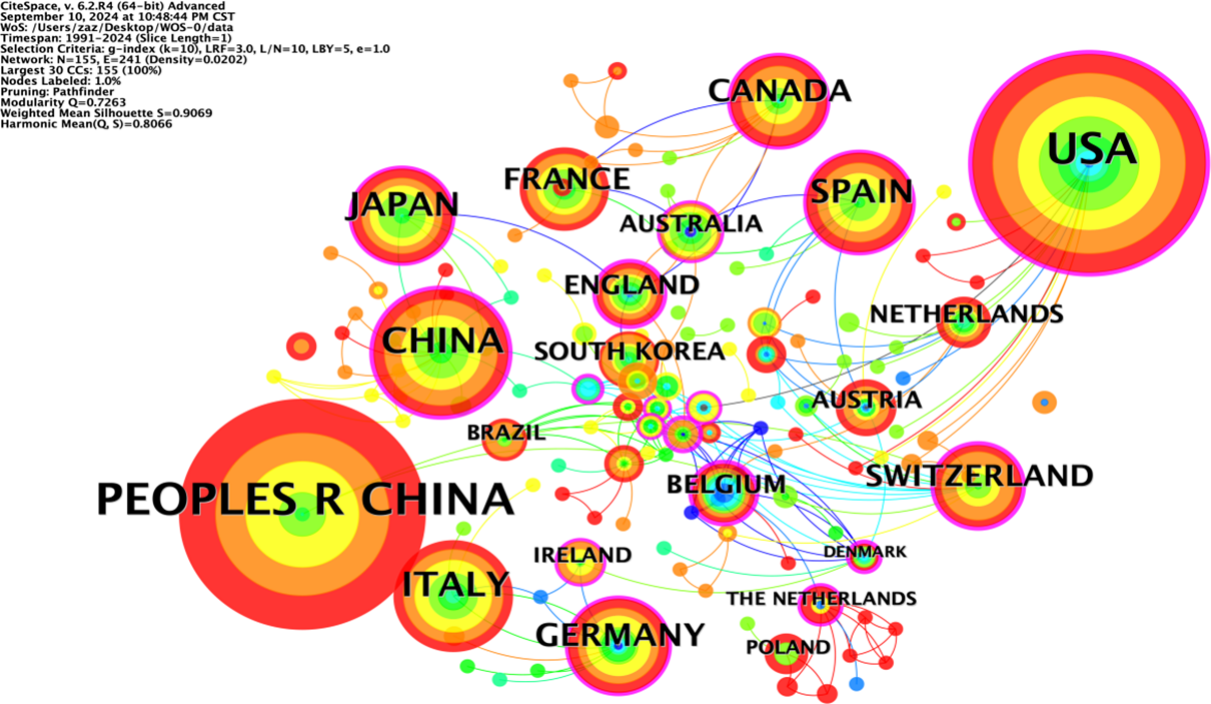
National co-occurrence map.

The co-occurrence network diagram of the national publication volume of research literature in the field of NIT for lung cancer (**Figure 3**) shows that the larger the radius of the node is, the higher the publication volume, and the thicker the peripheral ring is, the higher the centrality. Among the top ten countries in terms of publication volume in the co-occurrence chart, China (PEOPLES R CHINA) has the highest centrality (0.32), followed by the United Kingdom (0.27), the United States (0.25), and Switzerland (0.24). This indicates that China has a high centrality and a dominant position in the research field of NIT for lung cancer, and this high centrality is positively correlated with a high incidence of literature. China started its research in this field relatively late, with the earliest publication date being 2012. However, under the guidance of national policies and the promotion of scientific innovation, China’s achievements in the research of NIT for lung cancer have gradually increased in recent years, ranking first internationally with an annual publication of 387 articles. The United States ranks second, with 267 articles published annually.

In total, 151 countries/regions were found to actively participate in research in this field. At least ten or more publications from 17 countries/regions were included in the analysis. **Table 1** shows the top 10 countries/regions in terms of publication rankings; the top three countries/regions are China (n=387), the United States (n=267), and Italy (n=73). The cooperation network of these countries/regions is shown in **Figure 2**. The scope of cooperation between the United States and China is more comprehensive than that between other countries/regions, with major partners including Japan, Germany, Italy, France, and Canada, indicating that China and the United States are leading countries in researching new adjuvant immunotherapies for NSCLC.

**Table 1:** Top ten countries in terms of publication volume.

Rank	Count	Centrality	Year	Country
1	387	0.32	2012	PEOPLES R CHINA
2	267	0.25	1994	USA
3	73	0.04	2006	ITALY
4	65	0.14	2008	JAPAN
5	55	0.14	2016	SPAIN
6	51	0.12	2010	GERMANY
7	46	0.14	2016	CANADA
8	42	0.05	2008	FRANCE
9	38	0.24	2018	SWITZERLAND
10	24	0.27	2016	ENGLAND

### Analysis of the institutional co-occurrence chart

The node type was set to “Institution”; the threshold to “Top N=50”, k=10; the time interval to 1991-2024; and the time length partition to 1 y. The “GO” button was clicked to run the software and generate a co-occurrence chart of institutions. The top ten foreign research institutions in the field of NIT for NSCLC in the literature data are shown in **Table 2**, with the Department of Thoracic Surgery in the first tier, the Department of Medical Oncology in the second tier, the Department of Oncology in the third tier, the University of Texas System in the fourth tier, and other institutions in the fifth tier.

**Table 2:** Top 10 institutions in terms of publication volume.

Rank	Count	Centrality	Year	Institution
1	67	0.04	2019	Department of Thoracic Surgery
2	46	0.05	2020	Department of Medical Oncology
3	40	0.01	2018	Department of Oncology
4	38	0.06	2018	University of Texas System
5	37	0.03	2019	Chinese Academy of Medical Sciences - Peking Union Medical College
6	36	0.06	2018	UTMD Anderson Cancer Center
7	32	0.01	2019	Peking Union Medical College
8	31	0.03	2020	Shanghai Jiao Tong University
9	31	0.06	2021	Tianjin Medical University
10	29	0.28	2017	Department of Radiation Oncology

**Figure 4:**
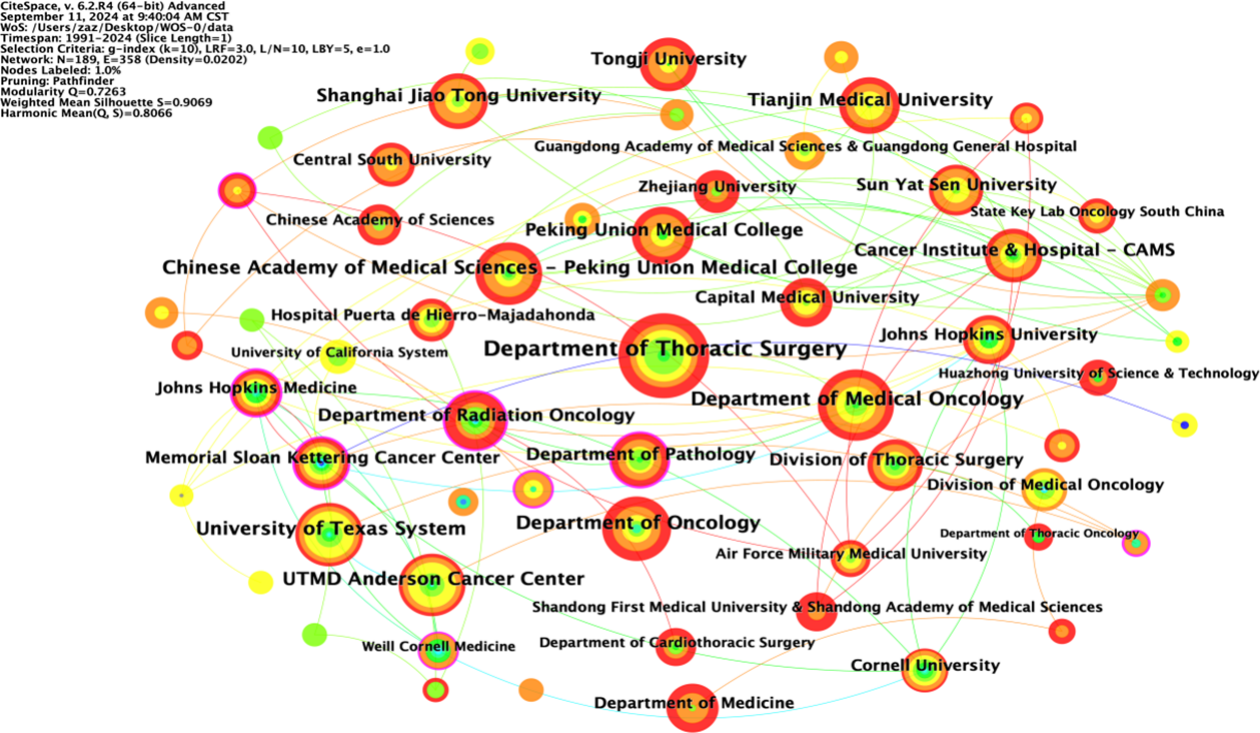
Co-occurrence of institutions.

The included English literature represented research at 189 institutions, with 40 institutions (21.2%) publishing ten or more articles and 18 institutions (9.5%) publishing 5-10 articles. The collaborative network of institutions publishing English language articles in the field of NIT for lung cancer is shown in **Figure 4**; 189 nodes and 358 lines were obtained, with a graph density of 0.0202, indicating relatively high cooperation between institutions. There were ten institutions publishing more than 29 articles, namely, the Department of Thoracic Surgery (67 articles), Department of Medical Oncology (46 articles), Department of Oncology (40 articles), University of Texas System (38 articles), Chinese Academy of Medical Sciences Peking Union Medical College (37 articles), UTMD Anderson Cancer Center (36 articles), Peking Union Medical College (32 articles), Shanghai Jiao Tong University, Tianjin Medical University (31 articles), and Department of Radiation Oncology (29 articles). Among these 10 institutions, 5 are Chinese universities, with the Department of Thoracic Surgery (Beijing Medical Association Thoracic Surgery Society) having the highest number of publications. This indicates that China is in a leading country in terms of number of institutions researching NIT for lung cancer; however, its centrality is relatively low, and its overall international influence still needs to be strengthened.

### Author chart

**Figure 5:**
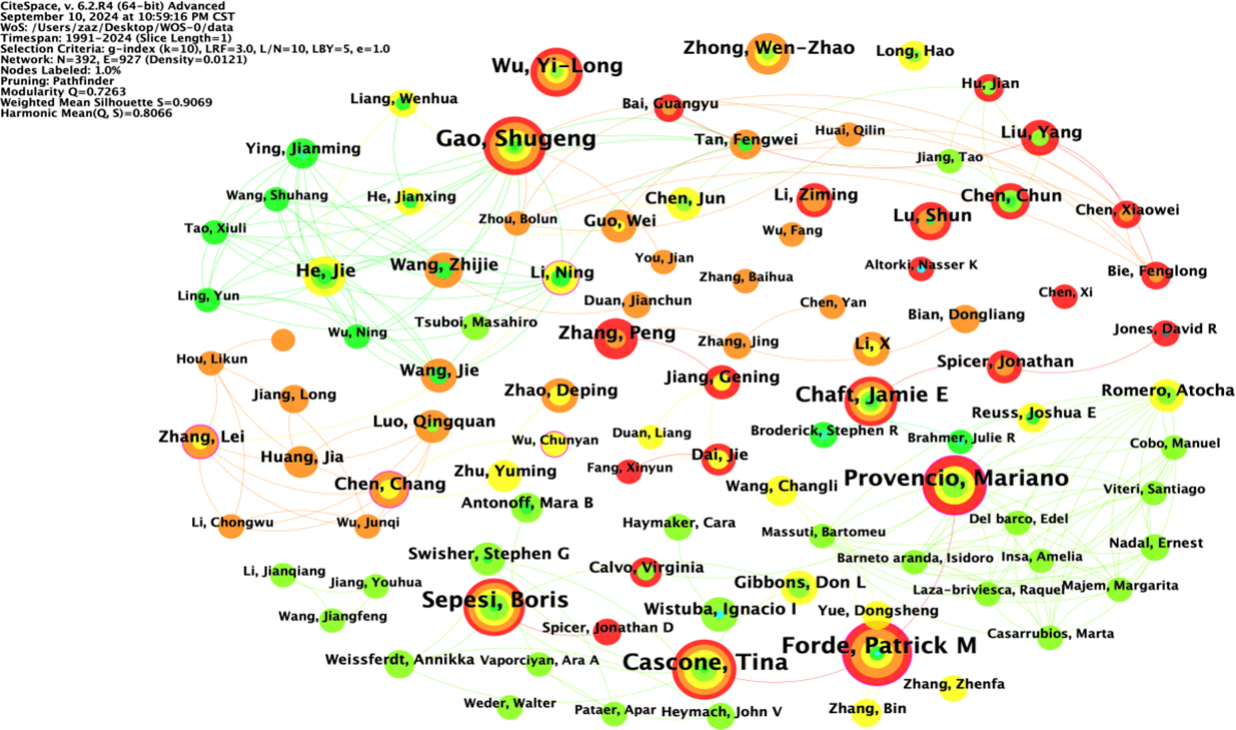
Author collaboration network diagram.

The node type was set to “Author”; the threshold to “Top N=50”, k=10; the time interval to 1991-2024; and the time length partition to 1 y. The “GO” button was clicked to run the software and draw the author collaboration network graph. Taking the author as the view node, N=392 in the figure, E=927, and density=0.0121. There were 392 authors included in the view, with 927 collaborative relationships between authors, forming a foreign author collaborative knowledge graph with a network density of 0.0121, as shown in **Figure 5**. The size of nodes and fonts in the network is directly proportional to the number of articles published by the author. The 1575 English studies included 392 authors, with 33 authors (8.4%) publishing ten or more articles and 88 authors (22.4%) publishing 5-10 articles. Visual analysis of the collaboration network was conducted on the authors (**Figure 3**), and it was found that the teams centered around GaoShugeng, Provencio, Mariano, and others had the highest number of publications and close connections between teams, forming an excellent collaborative network.

The top ten authors in the field of NIT for lung cancer in terms of publication volume are shown in **Table 3**. Among them, Forde and Patrick M are in the first tier, with a publication volume of 40 articles. Cascone and Tina are in the second tier, with 37 published articles. GaoShugeng, Provencio, and Mariano are in the third tier, with 35 published articles. The other authors are in the fourth tier. From the perspective of foreign authors’ publication and cooperation, the overall publication situation is relatively average. Nevertheless, there is relatively little cooperation among authors, and a widely connected cooperative relationship has yet to be formed. More cooperative research in the field of NIT for NSCLC should be strengthened to promote the development of this field jointly.

**Table 3:** Top ten authors in terms of publication volume.

Rank	Count	Centrality	Year	Author
1	40	0.15	2018	Forde, Patrick M (12)
2	37	0.07	2020	Cascone, Tina (13)
3	35	0.03	2020	Gao, Shugeng (14)
4	35	0.2	2020	Provencio, Mariano (15)
5	34	0	2020	Sepesi, Boris (16)
6	26	0.02	2018	Chaft, Jamie E (17)
7	24	0.01	2021	Wu, Yi-Long (18)
8	17	0.07	2023	Zhang, Peng (19)
9	17	0	2021	Zhong, Wen-Zhao (20)
10	16	0.07	2020	He, Jie (21)

### Co-citation analysis of the literature

**Figure 6:**
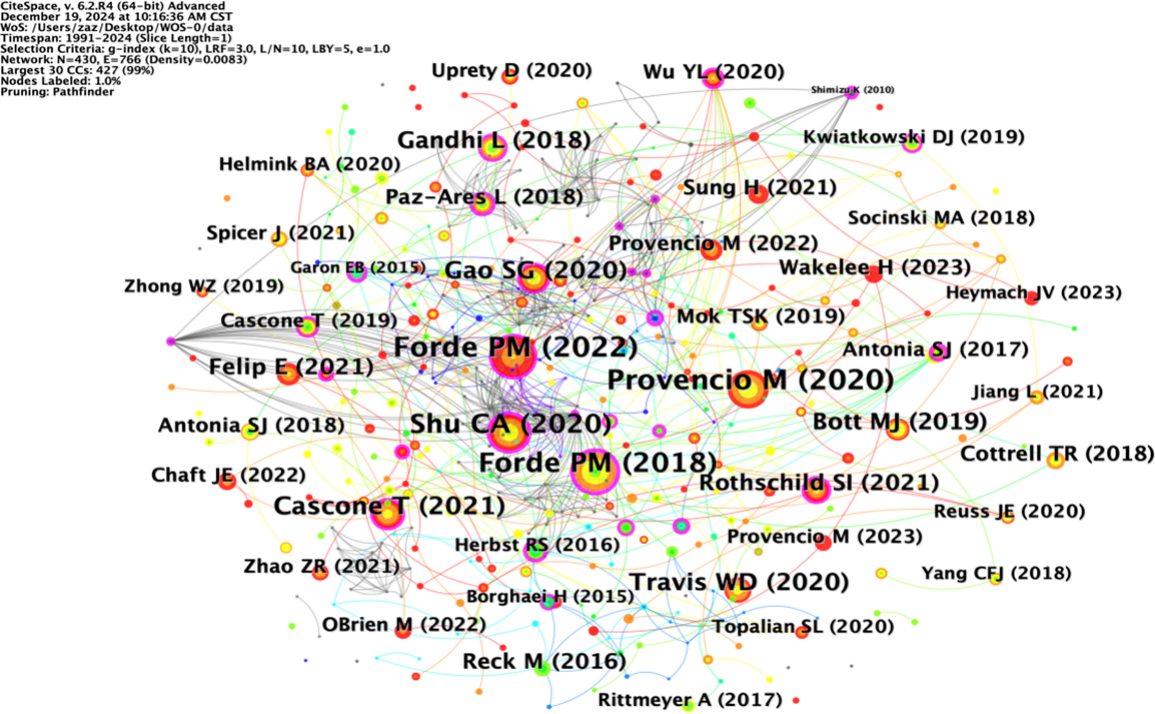
Diagram of the literature co-citation analysis.

Co-citation analysis can analyze the research hotspots in a specific research field. **Table 4** lists the top ten most co-cited studies in the field of neoadjuvant immunotherapy for lung cancer. Exploring the theoretical basis of these studies helps us understand the important information in this field. The most frequently co-cited article is the paper titled “Neoadjuvant nivolumab plus chemotherapy in resectable lung cancer” written by Forde PM and published in “NEW ENGL J MED” in 2022, with a citation frequency of 184. This article provides important evidence for the efficacy and safety of nivolumab combined with chemotherapy as a neoadjuvant treatment for resectable NSCLC and supports its use as a treatment option. The second most co-cited paper is titled “Neoadjuvant chemotherapy and nivolumab in resectable non-small-cell lung cancer (NADIM): an open-label, multicenter, single-arm, phase 2 trial” written by Provencio M and published in “NEW ENGL J MED” in 2020, with a citation frequency of 176. This article suggests that neoadjuvant chemoimmunotherapy may change the perception of locally advanced lung cancer as a potentially fatal disease and make it curable. The long-term follow-up results further confirm the long-term benefits of neoadjuvant chemoimmunotherapy without concerning safety data, strengthening its use in patients with resectable stage IIIA NSCLC. Although the above two articles have different research focuses, they both provide important insights and form an theoretical basis for research in this field (**Figure 6**).

**Table 4:** Top 10 cited articles.

Rank	Count	Centrality	Year	Cited article
1	184	0.12	2022	Forde PM, 2022, NEW ENGL J MED, V386, P1973, DOI 10.1056/NEJMoa2202170
2	176	0	2020	Provencio M, 2020, LANCET ONCOL, V21, P1413, DOI 10.1016/S1470-2045(20)30453-8
3	175	0.58	2018	Forde PM, 2018, NEW ENGL J MED, V378, P1976, DOI 10.1056/NEJMoa1716078
4	142	0.37	2020	Shu CA, 2020, LANCET ONCOL, V21, P786, DOI 10.1016/S1470-2045(20)30140-6
5	94	0.14	2021	Cascone T, 2021, NAT MED, V27, P504, DOI 10.1038/s41591-020-01224-2
6	88	0.12	2020	Gao SG, 2020, J THORAC ONCOL, V15, P816, DOI 10.1016/j.jtho.2020.01.017
7	79	0	2020	Travis WD, 2020, J THORAC ONCOL, V15, P709, DOI 10.1016/j.jtho.2020.01.005
8	74	0.19	2018	Gandhi L, 2018, NEW ENGL J MED, V378, P2078, DOI 10.1056/NEJMoa1801005
9	65	0.03	2019	Bott MJ, 2019, J THORAC CARDIOV SUR, V158, P269, DOI 10.1016/j.jtcvs.2018.11.124
10	61	0.02	2021	Felip E, 2021, LANCET, V398, P1344, DOI 10.1016/S0140-6736(21)02098-5

### Keyword co-occurrence analysis

Keywords are highly concise summaries of the content of an article, enabling readers to quickly understand the research topic. The node type was set to “keyword”; the threshold to “Top N=50”, k=10; the time interval to 1991-2024; and the time length partition to 1 year. The “GO” button was clicked to run the software, and the keyword co-occurrence graph was adjusted for visual effect. CiteSpace extracted keywords from relevant articles in the field of NIT for lung cancer from the Web of Science, PubMed, and Scopus databases to form a co-occurrence map of keywords, which can intuitively reflect the research topics in this field.

**Figure 7:**
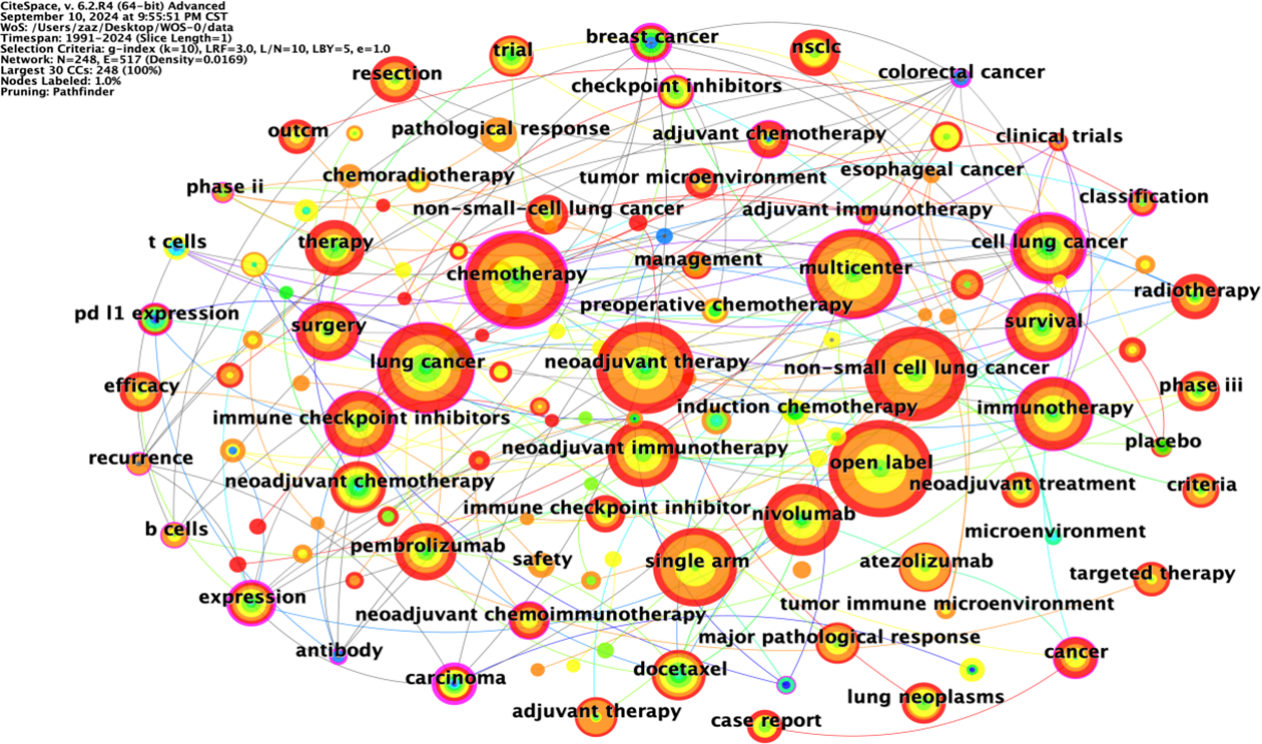
Keyword co-occurrence chart.

The co-occurrence map of literature keywords in the field of NIT for lung cancer consists of 248 nodes and 517 lines (**Figure 7**). The most central keyword among them is cell lung cancer, which highlights the attention given by other countries to NSCLC research. According to the analysis of the co-occurrence graph of keywords, the top 10 keywords in the literature, ranked in descending order of word frequency, are open-label chemotherapy, non-small cell lung cancer, lung cancer, neoadjuvant therapy, multicenter, single-arm, immunotherapy, nivolumab, and cell lung cancer (**Table 5**). Compared with those of other keywords, the co-occurrence frequencies of the above keywords are relatively high. Intermediary centrality is a crucial indicator for evaluating the importance of network nodes. In the co-occurrence network knowledge graph of keywords in the field of lung cancer neoadjuvant immunotherapy, keywords with high centrality are cell lung cancer (0.38), chemotherapy (0.2), immunotherapy (0.18), lung cancer (0.15), and multicenter (0.13), indicating that cell lung cancer, chemotherapy, immunotherapy, lung cancer, and multicenter are research hotspots in the field of lung cancer neoadjuvant immunotherapy. Among these keywords, immunotherapy accounts for the majority.

**Table 5:** Statistics of high-frequency keywords and centrality.

Rank	Count	Centrality	Keyword
1	170	0.08	open-label
2	161	0.2	chemotherapy
3	160	0.04	non-small cell lung cancer
4	153	0.15	lung cancer
5	138	0	neoadjuvant therapy
6	128	0.13	multicenter
7	101	0.02	single arm
8	96	0.18	immunotherapy
9	93	0.08	nivolumab
10	91	0.38	cell lung cancer

### Keyword clustering analysis

**Figure 8:**
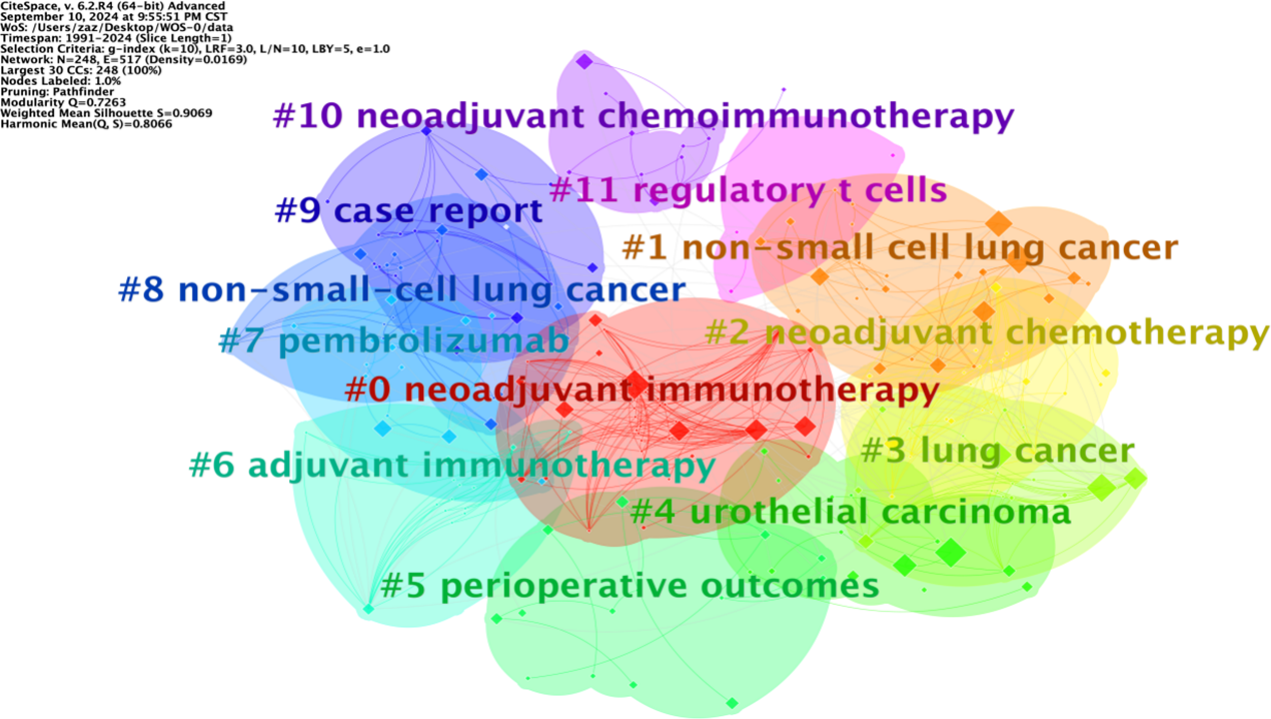
Keyword clustering diagram.

Keyword clustering analysis is based on the co-occurrence of keywords in literature. It uses clustering statistical methods to simplify the network relationship of keyword co-occurrence into several relatively few clustering processes, which helps researchers discover the hotspot research topics in the academic community. Based on keyword co-occurrence in CiteSpace, this article uses the LLR algorithm to obtain a clustering map (**Figure 7**) and statistically analyzes the data in the keyword clustering map to obtain a clustering information table. The high-frequency keyword analysis results show that there are currently 12 main clusters. The clustering results are shown in **Table 6**. The keyword cluster labels include # 0 neoadjuvant immunotherapy, # 1 non-small cell lung cancer, # 2 neoadjuvant chemotherapy, # 3 lung cancer, # 4 urological carcinoma, # 5 periodic outcomes, # 6 adjuvant immunotherapy, # 7 pembrolizumab, # 8 non-small cell lung cancer, # 9 case report, # 10 neoadjuvant chemoimmunotherapy, and # 11 regulatory T cells. The contour values of each cluster are all >0.8, indicating high clustering consistency and good homogeneity results. Therefore, published research on lung cancer is based mostly on neoadjuvant immunotherapy, neoadjuvant chemotherapy, and other methods (**Figure 8**).

**Table 6:** Keyword clustering information.

Rank	Cluster label	Scale value	Contour value	Main keywords
1	#0 neoadjuvant immunotherapy	31	0.875	neoadjuvant immunotherapy; immune checkpoint inhibitors; PD-L1; PD-1; neoadjuvant treatment
2	#1 non-small cell lung cancer	26	0.985	non-small cell lung cancer; neoadjuvant treatment; immune checkpoint inhibitors; lung cancer; immune-related adverse events
3	#2 neoadjuvant chemotherapy	22	0.935	neoadjuvant chemotherapy; breast cancer; rectal cancer; esophageal cancer; ovarian cancer
4	#3 lung cancer	21	0.878	lung cancer; neoadjuvant therapy; cancer immunotherapy; targeted drug delivery; complications
5	#4 urothelial carcinoma	20	0.987	urothelial carcinoma; survival; CTLA-4; targeted therapy; pneumonectomy
6	#5 perioperative outcomes	17	0.808	perioperative outcomes; early-stage NSCLC; EGFR; ALK; IIIA (N2) disease
7	#6 adjuvant immunotherapy	16	0.948	adjuvant immunotherapy; thymoma; disseminated tumor cells; spontaneous remission; consensus
8	#7 pembrolizumab	16	0.843	pembrolizumab; tumor microenvironment; NSCLC; atezolizumab; tumor mutation burden
9	#8 non-small cell lung cancer	16	0.919	non-small cell lung cancer; lung neoplasms; adjuvant chemotherapy; united states food and drug administration; humans
10	#9 case report	15	0.961	case report; conversion treatment; immunotherapy neoadjuvant treatment; N3 locally advanced NSCLC; dendritic cells
11	#10 neoadjuvant chemoimmunotherapy	14	0.929	neoadjuvant chemoimmunotherapy; immune checkpoint inhibitors; major pathologic response; clinical trial; adverse events
12	#11 regulatory T cells	14	0.843	regulatory T cells; tertiary lymphoid structures; translational medical research; tumor biomarkers; IgA

### Keyword timeline analysis

**Figure 9:**
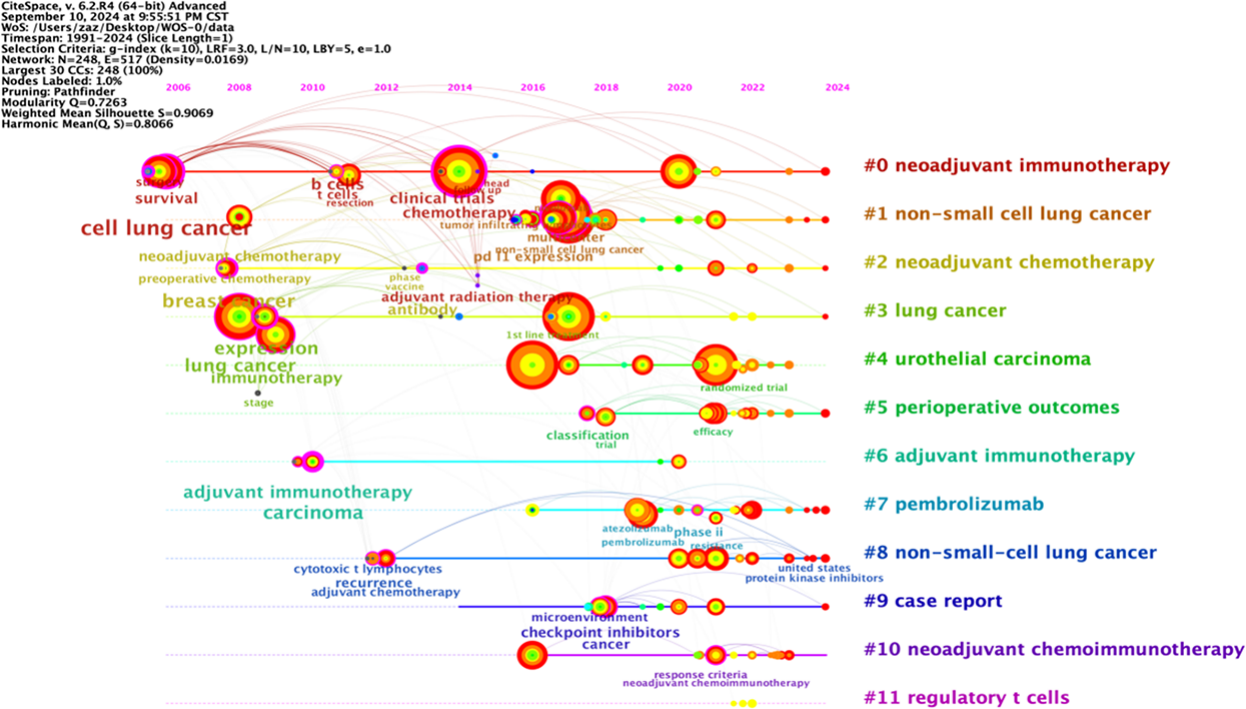
Timeline of keyword clustering.

The keyword graph from 1991 to 2024 was converted into a timeline clustering graph (**Figure 9**). Overall, # 0 neoadjuvant immunotherapy, # 1 non-small cell lung cancer, and # 2 neoadjuvant chemotherapy have an extended period and a relatively large number of publications, making them long-term research directions. Clusters # 7 pembrolizumab and # 11 regulatory T cells have a short period and low publication volume. Additional studies and more in-depth research are necessary.

The longitudinal analysis of the timeline clustering timeline can be roughly divided into two stages. The first stage (2002-2013) is the initial development stage of NIT for lung cancer, which focuses on research in cell lung cancer, breast cancer, lung cancer, and adjuvant immunotherapy. The second stage (from 2014 to 2024) involves rapid development in lung cancer research. In this stage, research gradually shifts to aspects such as chemotherapy, non-small cell lung cancer, cancer, and neoadjuvant chemoimmunotherapy.

In horizontal analysis, the popularity of the research continued to increase between 2014 and 2024, and there were many connections between keywords, including intracluster and cross-cluster connections. This indicates that research during this period was relatively concentrated and had a high level of enthusiasm, which could be combined with various fields and indirectly reflect the research trends in this field in the coming years.

### Keyword emergence analysis

**Figure 10:**
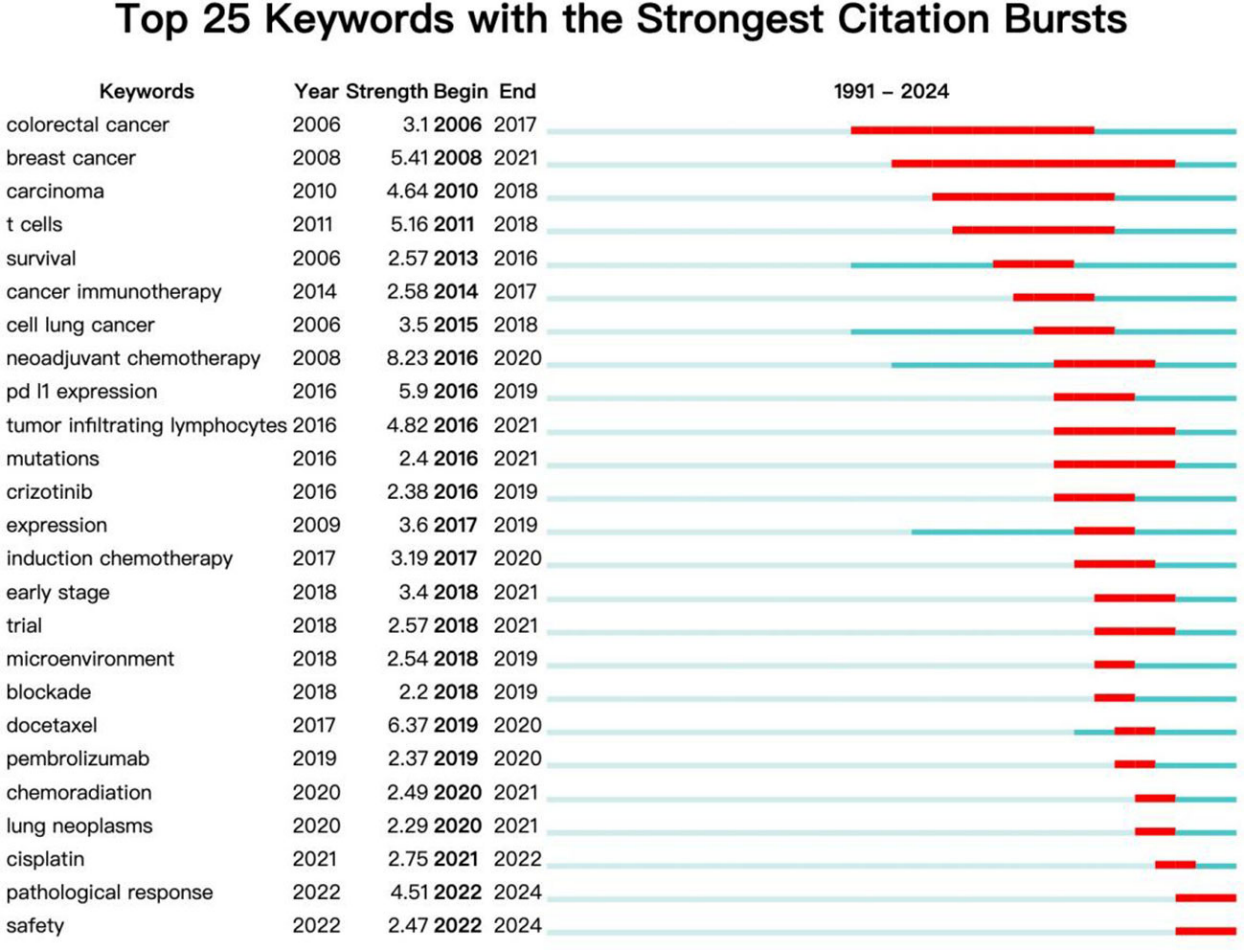
Keyword emergence chart.

The keyword burst detection function of CiteSpace can analyze keywords that have undergone significant changes in the short term and display the start and end years and mutation intensity of the keyword. It can obtain past and current research hotspots from the keyword’s perspective. The term ‘begin’ represents the time when the keyword suddenly appears, ‘end’ represents its end time, and ‘strength’ represents the strength of the keyword mutation. The keyword burst analysis of the literature on NIT for lung cancer is shown in **Figure 10**. In terms of salience, the top 5 salience words are neoadjuvant chemotherapy (8.23), docetaxel (6.37), PD-L1 expression (5.9), breast cancer (5.41), and tumor inflammatory lymphocytes (4.82). From the perspective of research duration, research before 2014 focused mainly on colorectal cancer, breast cancer, cancer, T cells, and other aspects (breakthrough cancer and survival); research hotspots after 2014 focused mainly on cancer immunotherapy, cell lung cancer, neoadjuvant chemotherapy, tumor-infiltrating lymphocytes, mutations, induction chemotherapy, paclitaxel, pembrolizumab, chemoradiation, lung cancer, pathological response, safety and other aspects (docetaxel). Among these areas of focus, research on neoadjuvant chemotherapy is relatively active, with the highest burst intensity among all burst words (8.23). The above results indicate that cancer immunotherapy, neoadjuvant chemotherapy, pathological response, and safety may become future hotspots in research on NIT for lung cancer.

### Dual-map overlay analysis of journals

**Figure 11:**
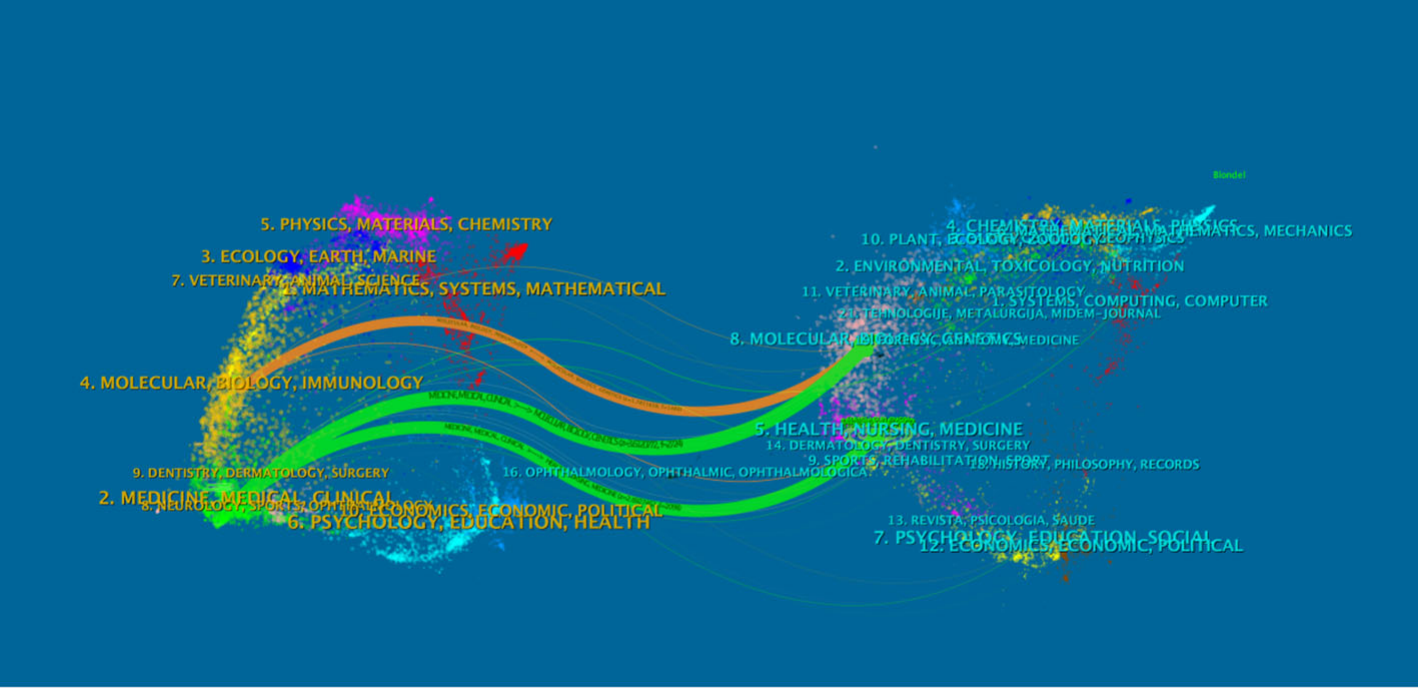
Dual-map overlay.

As an advanced function of CiteSpace, the dual-map overlay of journals (**Figure 11**) can display information such as the distribution of papers in various disciplines, citation trajectories, and center-of-gravity drift. In the dual-map overlay result of journals, the left side is the citing map, and the right side is the cited map. The curve is the citation link, which ultimately shows the context of citations. In the left graph, the more papers a journal publishes, the longer the vertical axis of the ellipse is, and the more authors there are, the longer the horizontal axis of the ellipse is.

**Figure 12:**
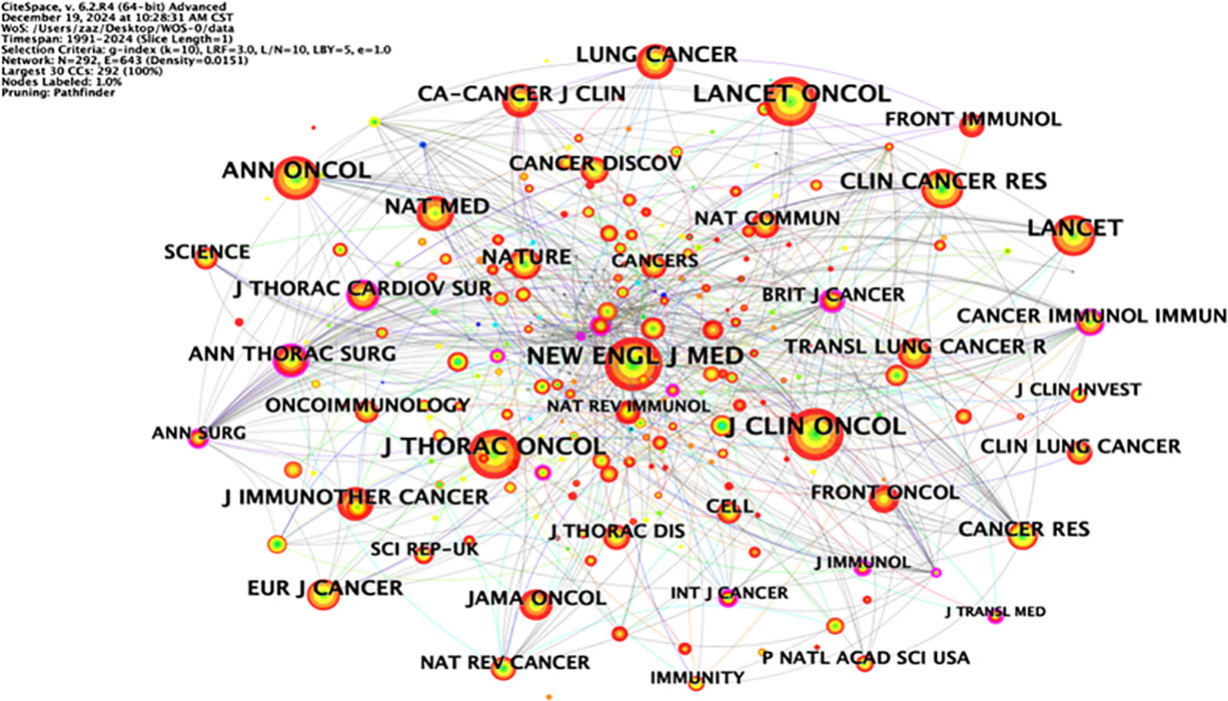
Visualization map of journals..

The labels on the left side of the map represent the disciplines of the cited journals, and the labels on the right side represent the disciplines of the journals in which the cited papers are published. Most articles are published in molecular biology, immunology, medicine, and surgery journals. In addition, most publications are cited in journals related to molecular biology, environment, nutrition, health care, health, rehabilitation, medicine, and psychology. **Figure 12** shows the visualization map of journals.

The following table lists the top 20 journals, which can be used to understand the distribution of research topics in this research field. Among them, the journals with a total number of published articles exceeding 300 are “NEW ENGL J MED” (469 articles), “J CLIN ONCOL” (463 articles), “J THORAC ONCOL” (437 articles), “LANCET ONCOL” (393 articles), “ANN ONCOL” (325 articles), and “LANCET” (302 articles). The abovementioned journals are important carriers of articles in this field and represent the key research directions (**Table 7**).

**Table 7:** Statistics of the top 20 journals.

Rank	Count	Centrality	Year	Journal
1	469	0.05	2010	NEW ENGL J MED
2	463	0.09	2008	J CLIN ONCOL
3	437	0.03	2011	J THORAC ONCOL
4	393	0.01	2010	LANCET ONCOL
5	325	0.06	2006	ANN ONCOL
6	302	0.07	2008	LANCET
7	286	0.08	2009	CLIN CANCER RES
8	226	0.08	2008	LUNG CANCER
9	215	0.05	2009	NAT MED
10	200	0.02	2006	CA-CANCER J CLIN
11	189	0.07	2017	J IMMUNOTHER CANCER
12	183	0.02	2006	EUR J CANCER
13	181	0.04	2016	NATURE
14	164	0.11	2010	ANN THORAC SURG
15	158	0.1	2006	CANCER RES
16	153	0.02	2018	JAMA ONCOL
17	150	0	2017	TRANSL LUNG CANCER R
18	147	0.04	2018	CANCER DISCOV
19	145	0.15	2011	J THORAC CARDIOV SUR
20	128	0	2020	FRONT ONCOL

### Analysis of associated gene clustering

**Figure 13:**
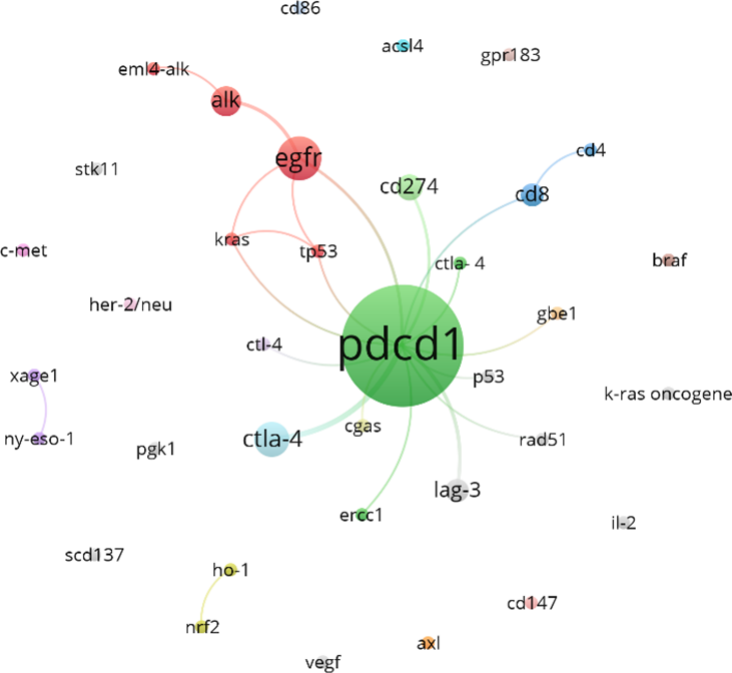
Clustering analysis of genes related to lung cancer and neoadjuvant immunotherapy.

**Figure 13** shows the gene clustering analysis related to lung cancer and neoadjuvant immunotherapy during this period, highlighting that PDCD1 is the gene most frequently mentioned in the literature (82 articles). EGFR and CTLA-4 follow closely behind, with 11 and 7 mentions, respectively.

### Subgroup analysis

**Table 8** shows the subgroup analysis for major pathological response (MPR), with data sourced from nine trials. Overall, in most subgroups, the OR value was greater than 1, and the 95% confidence interval did not contain 1, indicating that the probability of events occurring in the treatment group was greater and that the treatment might be effective. Moreover, the I² value of most subgroups was low, suggesting a high degree of consistency in the research results and high credibility. Among them, the treatment effect in the <65-year-old group was better than that in the ≥65-year-old group [OR: 7.03 > 3.22]. There were differences between the female and male groups, different regional groups, different histological type groups, different frequency groups, different ECoG score groups, and different PD-L1 expression level groups. The current or former smoker group had statistically significant differences, whereas the never smoked group did not.

**Table 8:** Results of subgroup analysis for major pathological response.

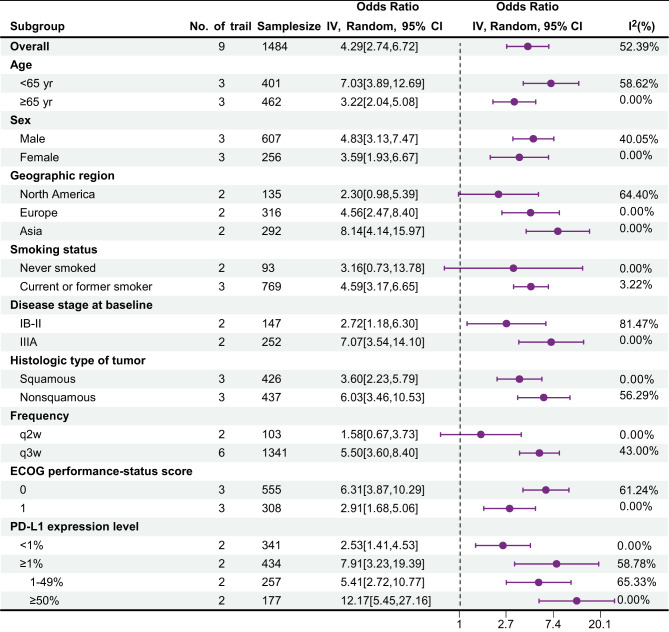

**Table 9** presents the subgroup analysis for pathological complete response (pCR), with data obtained from nine trials. Overall, in most subgroups, the OR value was greater than 1, and the 95% confidence interval did not contain 1, indicating that the probability of events occurring in the treatment group was great and that the treatment might be effective. Moreover, the I² value of most subgroups was low, suggesting a high degree of consistency in the research results and high credibility. Specifically, the treatment effect in the <65-year-old group was better than that in the ≥65-year-old group [OR: 14.33 > 6.04]. There were differences between the female and male groups, different regional groups, different disease stage groups, different histological type groups, different frequency groups, different ECoG score groups, and different PD-L1 expression level groups. The current or former smoker group had statistically significant differences, whereas the never smoked group did not. The forest plot illustrates the treatment effects and statistical significance of each subgroup.

**Table 9:** Results of subgroup analysis for pathological complete response.

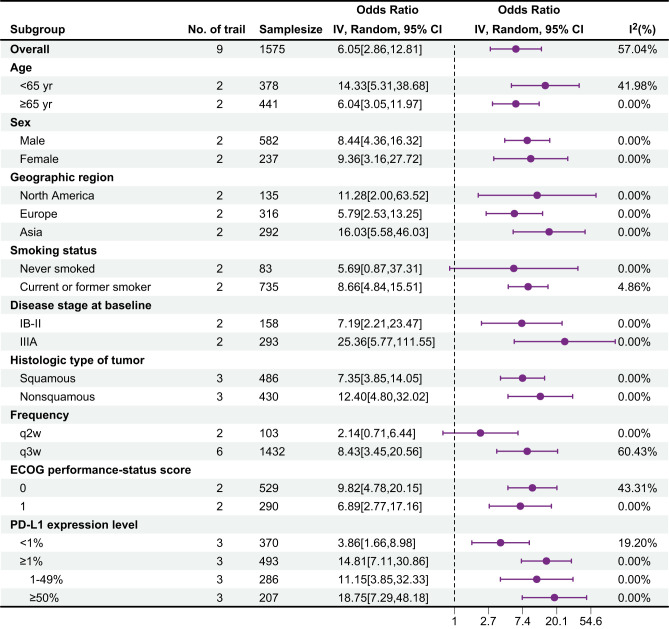

## Discussion

In the past decade, there has been a significant shift in the treatment of lung cancer, particularly for NSCLC. Immunotherapy, especially neoadjuvant immunotherapy, has become an essential option for patients. We systematically and objectively describe the trends in NIT for lung cancer and identify future research hotspots to assist scholars in quickly understanding the research landscape in this field and to provide valuable guidance for topic selection activities.

The first part of this study analyzes publishing trends, countries, institutions, authors, and journals. We subsequently conduct a clustering analysis of keywords to identify research hotspots within the field.

According to the publishing trend analysis results, the number of publications on NIT for lung cancer has rapidly increased over the past decade, reaching a record high of 349 articles in 2023, with expectations for further growth in 2024. This indicates that the field is garnering increasing attention, and the continuously rising trend underscores the important position of this field in international research.

A total of 151 countries/regions around the world have actively participated in research on the application neoadjuvant immunotherapy for lung cancer. Among them, China has the highest annual number of publications, with 387 articles, and a high centrality of 0.32, followed by the United States. There are significant differences among countries in terms of the number of publications, centrality, and starting time. China has made remarkable progress in this field. Through multiple clinical studies, such as CheckMate-159 (12), the NADIM series (22), and CheckMate-816 (23), the effectiveness of various treatment modalities, such as single-agent immunotherapy, combined chemotherapy, dual immunotherapy, and neoadjuvant combined with adjuvant therapy, has been confirmed. Nivolumab combined with platinum-based doublet chemotherapy has been approved for specific patients, and atezolizumab and others also show good potential. The United States has also made outstanding achievements in this field. Its research is not only large in volume and scale but also in depth. Studies such as CheckMate-816 (23) have analyzed surgical-related indicators in detail and have continuously carried out more research on new treatment modalities. Multiple immune checkpoint inhibitors have been approved for application first, and they have significant advantages in biomarker research and application, the degree of multidisciplinary collaboration, and other aspects. However, compared with the United States, China still has certain gaps in the number and scale of clinical studies; the depth and breadth of research; the speed of drug research, development and approval; the transformation of biomarker research results; and the degree of multidisciplinary collaboration. This also provides many inspirations for subsequent international research. In terms of resource investment and policy guidance, other countries can learn from China’s experience and optimize resource allocation according to their own national conditions. In terms of international cooperation, all countries should actively expand cooperation to achieve complementary advantages, and weaker countries should take the initiative to integrate. As part of the scientific research development model, different countries and research teams should learn from each other, improve management and cultivate talent to promote continuous in-depth development in the field of neoadjuvant immunotherapy for lung cancer and in medical research as a whole.

Among the authors investigated, Patrick M. Forde is the leading contributor, with 40 publications, followed by Tina Cascone, who has 37 publications.

In our thematic analysis and mapping, we identified keywords such as “cancer immunotherapy,” “neoadjuvant chemotherapy,” “pathological response,” and “safety” as potential future hotspot research topics. A co-occurrence analysis of these keywords revealed five clusters, each representing distinct research trends and hotspots, which we discuss in detail.

This review thoroughly elucidates the current status of NIT research in non-small cell lung cancer, highlighting global contributions, influential institutions, authors, and future trends. These insights will assist researchers in better navigating the field, shaping future research directions, and improving treatment outcomes for lung cancer patients. This bibliometric analysis identified several critical subtopics related to NIT for non-small cell lung cancer.

### In-depth understanding of the mechanisms of immunotherapy

Immunotherapy combats tumors by activating the immune system and relieving immune suppression. Immune checkpoint inhibitors, especially programmed cell death protein 1 (PD-1), programmed cell death ligand 1 (PD-L1), and cytotoxic T-lymphocyte-associated protein 4 (CTLA-4), target the escape mechanisms of tumor cells (24). The binding of PD-1 on tumor cells to PD-L1 can inhibit the activation of immune cells. PD-1/PD-L1 inhibitors are able to revitalize the immune response against tumors (25). CTLA-4 suppresses the immune system by competing with CD28 for binding to B7 ligands and is expressed on regulatory T cells (26). Ipilimumab can prevent this inhibition and activate CD8^+^ T cells, which can kill tumor cells. The degree of infiltration of CD8^+^ tumor-infiltrating lymphocytes (TILs) is a crucial predictor of the efficacy of immunotherapy and patient prognosis and helps in the precise selection of appropriate treatment regimens for personalized medicine (27).

### Clinical studies and efficacy evaluation of neoadjuvant immunotherapy

In the field of neoadjuvant therapy for non-small cell lung cancer (NSCLC), various immunotherapy drugs are highly important. Pembrolizumab and nivolumab are both anti-PD-1 monoclonal antibodies that can activate T cells to inhibit tumors by blocking the binding of PD-1 to PD-L1. For example, as reported by Wakelee H et al. (28), neoadjuvant pembrolizumab combined with chemotherapy in patients with resectable stage II-IIIB NSCLC significantly improved the event-free survival (EFS), major pathological response (MPR) and pathological complete response (pCR) rates, with the EFS rate reaching 62.4%, the MPR rate reaching 30.2%, and the pCR rate reaching 18.1%. In the KEYNOTE-671 trial, neoadjuvant pembrolizumab combined with chemotherapy followed by adjuvant pembrolizumab was administered to patients with resectable stage II, IIIA, or IIIB (N2) NSCLC. The 36-month overall survival rate (71% in the pembrolizumab group and 64% in the placebo group) and the median event-free survival time (47.2 months in the pembrolizumab group and 18.3 months in the placebo group) were both better than those in the placebo group, and the safety was manageable (29). In the CheckMate-816 trial (23), nivolumab combined with chemotherapy in patients with resectable stage IB-IIIA NSCLC significantly increased the pCR rate (24.0 vs. 2.2%) and improved the EFS rate. In the CheckMate-77T trial (30), nivolumab combined with chemotherapy led to a better median EFS rate and a higher pCR rate in patients with resectable stage IIA-IIIB NSCLC than in the placebo group, while the rates among patients who received only an operation were similar.

Atezolizumab and durvalumab target PD-L1, which can restore T-cell function and influence the tumor microenvironment (31). In the LCMC3 trial (32), atezolizumab was administered as neoadjuvant treatment to patients with resectable stage IB-IIIA (partial stage IIIB) NSCLC. Eighty-eight percent of the patients underwent surgical resection, with 20.4% achieving major pathological response (MPR) and 6.8% achieving pathological complete response (pCR), and the safety was manageable. The AEGEAN trial demonstrated that durvalumab combined with chemotherapy could prolong event-free survival (EFS) and increase the pCR rate in patients with resectable stage II-IIIB (N2) NSCLC (33, 34). Camrelizumab exerts its antitumor effect by blocking the PD-1 pathway. The TD-FOREKNOW trial indicated that when camrelizumab was combined with chemotherapy and administered to patients with resectable stage IIIA or IIIB (T3N2) NSCLC, the pCR rate was 32.6% (35). Some bispecific antibody drugs are still in the research stage and are expected to lead to new breakthroughs in the administration of neoadjuvant immunotherapy for NSCLC (36, 37). In conclusion, although neoadjuvant immunotherapy for NSCLC has already shown potential, large-scale multicenter trials are still needed to explore and optimize the regimens.

### Potential of NIT combined with chemotherapy

The combination of immunotherapy and chemotherapy for the treatment of NSCLC is well known. Chemotherapy can kill tumor cells and induce immunogenic cell death to activate innate immunity, and immunotherapy (such as immune checkpoint inhibitors) can activate T cells, etc.; the two treatments interact to produce a synergistic antitumor effect. In the neoadjuvant treatment of NSCLC, the advantages of combined treatment are significant (38, 39). For example, in the CheckMate-816 study, the pCR rate in the nivolumab plus chemotherapy group reached 24%, which was approximately 11 times greater than that in the group receiving chemotherapy alone, and the median EFS was 31.6 months, nearly 11 months longer than that in the group receiving chemotherapy alone (23, 40). A prospective phase III study (41) divided patients with stage IIIB-IV nonsquamous non-small cell lung cancer (NSCLC) into a group treated with camrelizumab combined with chemotherapy and a group treated with chemotherapy alone. The median overall survival (OS) in the combined group reached 27.1 months, which was better than that of the group receiving chemotherapy alone (19.8 months). The objective response rate (ORR) reached 97.0%, and some of the responses persisted. In addition, the safety was manageable. In the CTONG1804 trial (42), a prospective phase II study divided patients with resectable stage IIA-IIIB NSCLC into a group receiving single-agent nivolumab (N) and a group receiving nivolumab plus chemotherapy (N/C). In the N/C group, the MPR rate reached 50.0%, the pCR rate reached 25.0%, and the 18-month EFS rate was 64.8%. The pCR rate of patients with PD-L1 expression ≥ 50% was 66.7%, which was higher than that of patients in the N group, and some indicators were correlated with PD-L1 expression and ctDNA status, highlighting the value of neoadjuvant immunotherapy combined with chemotherapy. The phase III trial (43) recruited patients with stage II/III resectable NSCLC (nonsquamous, EGFR/ALK-wild type). The patients were randomly divided into groups treated with toripalimab + chemo or placebo + chemo. The toripalimab group had significantly better event-free survival rate (not estimable vs. 15.1 months, HR = 0.40, P <0.001), major pathological response rate (48.5% vs. 8.4%, difference = 40.2%), and pathological complete response rate (24.8% vs. 1.0%, difference = 23.7%). The phase III RATIONALE-315 trial (44) enrolled patients with stage II-IIIA NSCLC and randomly divided them into a group treated with tislelizumab plus chemotherapy group and a group treated with placebo plus chemotherapy. The former had significantly better event-free survival rate (hazard ratio = 0.56, P = 0.0003) and major pathological response rate (56% vs. 15%, with a difference of 41%).

Neoadjuvant immunotherapy combined with chemotherapy can improve the pathological response rate, prolong survival time, and optimize surgery-related indicators and prognosis. This treatment combination can reduce the tumor volume before surgery, which is beneficial for surgery and improves the radical resection rate. After surgery, this treatment combination can maintain antitumor activity to prevent recurrence and metastasis and is not limited by driver gene mutations. Multimodal targeting of tumor cells can inhibit the development of drug resistance and result in more lasting curative effects for patients, optimizing the treatment strategy and overall prognosis in multiple aspects. Research (5–7, 22, 23, 28, 34, 40, 42–50) on neoadjuvant immunotherapy combined with chemotherapy in patients with NSCLC conducted within the past three years is listed in **Table 10**.

**Table 10:** Clinical research on neoadjuvant immunotherapy combined with chemotherapy for NSCLC.

Registration No.	Study	Phase	Number of cases	Patient stages	Neoadjuvant therapy group	ICI dose per cycle	Frequency	No. of cycles before surgery	Results	Reference
NCT03158129	NEOSTAR	II	44	IB-IIIA	Nivolumab + cisplatin (carboplatin) + pemetrexed vs. ipilimumab + nivolumab + cisplatin (carboplatin) + pemetrexed	360 mg + 75 mg/m2 (AUC 5/6) + 500 mg/m2 vs. 1 mg/kg (d1 only) + 360 mg + 75 mg/m2 (AUC 5/6) + 500 mg/m2	q3w	3	MPR 32.1 vs. 50%	(5)
NCT03081689	NADIM	II	46	IIIA	Paclitaxel + carboplatin + nivolumab	200 mg/m2 + AUC 6 + 360 mg	q3w	3	PFS (24 months) 77.1%, OS (36 months) 81.9%	(22)
NCT04015778	CTONG1804	II	52	IIA- IIIB	Nivolumab vs. nivolumab + nab-paclitaxel + carboplatin	360 mg vs. 360 mg + 135 mg/m2 (d1, d8) + AUC 5	q3w	3	MPR 50%, pCR 25%	(42)
NCT03838159	NADIM II	II	86	IIIA- IIIB	Nivolumab + paclitaxel + carboplatin vs. paclitaxel + carboplatin	360 mg + 200 mg/m2 + AUC 5 vs. 200 mg/m2 + AUC 5	q3w	3	pCR 37 vs. 7%, PFS (24 months) 67.2 vs. 40.9%, OS (24 months) 85.0 vs. 63.6%	(6)
NCT04326153	Neo- Pre-IC	II	20	IIIA- IIIB	Sintilimab + nab-paclitaxel + carboplatin	200 mg + 260 mg/m2 +AUC 5	q3w	2-3	ORR 55%, MPR 65%, pCR 40%	(7, 45)
ChiCTR 1900023758		II	50	IIIA	Sintilimab + carboplatin + gemcitabine	200 mg + AUC 5 + 1 000 mg/m2 (d1, d8)	q3w	2-4	MPR 43.3%	(46)
ChiCTR 1900024014	LungMate 002	II	50	II- III	Toripalimab + carboplatin-based chemotherapy	240 mg	q3w	2-4	ORR 76%, MPR 55.6%, pCR 27.8%	(47)
NCT02998528	Checkmate-816	III	505	IB- IIIA	Nivolumab + platinum-based chemotherapy vs. platinum- based chemotherapy	360 mg	q3w	3	EFS 31.6 vs. 20.8 months, pCR 24% vs. 2.2%	(23, 40)
NCT03800134	AEGEAN	III	802	II- IIIB	Durvalumab + platinum-based chemo- therapy vs. platinum-based chemotherapy	1500 mg	q3w	4	EFS (12 months) 73.4 vs. 64.5%, pCR 17.2 vs. 4.3%	(34, 48–50)
NCT03425643	KEYNOTE- 671	III	797	II- IIIB	Pembrolizumab + cisplatin + gemcitabine/pemetrexed vs. cisplatin + gemcitabine/pemetrexed	200 mg	q3w	4	EFS (24 months) 62.4 vs. 40.6%, OS (24 months) 80.9 vs. 77.6%, MPR 30.2 vs. 11.0%	(28)
NCT04158440	Neotorch	III	501	II- IIIB	Toripalimab + platinum-based chemo- therapy vs. platinum-based chemotherapy	240 mg	q3w	4	MPR 48.5 vs. 8.4%, pCR 24.8 vs. 1.0%, EFS > 24.4 vs. 15.1 months	(43)
NCTO4379635	RATIONALE- 315	III	453	II- IIIA	Tislelizumab + platinum-based chemo- therapy vs. platinum-based chemotherapy	200 mg	q3w	3-4	MPR 56.2 vs. 15.0%, pCR 40.7 vs. 5.7%	(44)
NCT03134872	CameL	III	412	IIIB - IV	Camrelizumab + pemetrexed vs. pemetrexed	200 mg	q3w	4-6	OS 27.1 vs. 19.8 months, ORR 79%	(41)

### Response to NIT and assessment of treatment efficacy

The response to NIT manifests in multiple dimensions. Clinically, there may be improvements in symptoms, whereas imaging studies may reveal changes in tumor size and metabolic activity, including pseudoprogression. Pathologically, the evaluation relies primarily on the MPR and pCR (51).

A variety of assessment methods are employed to evaluate treatment efficacy. Imaging assessments, in addition to traditional modalities such as CT, PET-CT, and MRI, benefit from the application of radiomics, which adds a new dimension to monitoring. Radiomics holds significant value in immunotherapy, as its features are closely linked to the immune cell infiltration status within the tumor microenvironment. Some imaging characteristics correlate with the activity and distribution of intratumoral T cells, providing novel pathways for assessing tumor sensitivity to immunotherapy (52). Quantitative comparisons can be made before and after treatment, effectively assisting clinicians in monitoring tumor changes and patient treatment responses. When combined with clinical data and molecular characteristics, radiomics can enhance the accuracy of prognostic predictions. Specific radiomic features associated with PD-L1 expression levels can aid in the precise selection of patients suitable for neoadjuvant immunotherapy, offering new posttreatment assessment methods by quantifying tumor shrinkage and pathologic response, thereby optimizing subsequent treatment plans (53).

As a noninvasive assessment tool, radiomics is widely applied in NSCLC immunotherapy, accurately predicting the expression of immune checkpoints and the composition of the tumor microenvironment while enabling real-time monitoring of treatment effects. It allows for noninvasive, dynamic evaluation of PD-L1 expression and treatment efficacy in NSCLC (54, 55). Related studies have demonstrated the role of CT texture analysis-based radiomic scoring and deep learning models in this context. In the field of predicting neoadjuvant treatment for non-small cell lung cancer, multiple studies have demonstrated the application and value of different strategies. The team led by Yun Lang She (56) collected 274 patients and calculated a deep learning score using the ShuffleNet v2 x0.5 features of the primary tumor in preoperative CT scans to predict the major pathological response (MPR). The areas under the curve (AUCs) in the internal and external cohorts reached 0.73 and 0.72, respectively, and the performance improved after combination with clinical features, highlighting the significance of radiomics in predicting neoadjuvant immunotherapy for lung cancer. Another team (57) collected 260 patients with stage IIA-IIIB disease and constructed radiomic and hybrid models to predict the MPR. The delta-radiomics model showed excellent AUC performance in multiple databases, and the hybrid model had a higher AUC, emphasizing the significant advantage of multimodal fusion of radiomics in accurate prediction and providing new ideas for clinically judging patients’ response to neoadjuvant chemoimmunotherapy. Additionally, a study (58) prospectively collected blood samples from 45 patients with stage IIIA (N2) disease and developed an integrated model by combining CT-based deep learning scores, blood tumor mutation burden, and clinical factors. The AUC of the improved DL model was 0.703, and that of the integrated model reached 0.820, reflecting the crucial role of the combination of radiomics and other factors in predicting the efficacy of neoadjuvant immunotherapy and strongly supporting clinical decision making.

Moreover, the assessment of immune-related biomarkers such as PD-L1 expression, TILs, circulating tumor cells (CTCs), and circulating tumor DNA (ctDNA) can reflect the immune status and tumor cell changes (59–61). In addition to immune function tests and quality-of-life assessments, the comprehensive use of these evaluation methods enables a thorough and accurate judgment of treatment efficacy, providing a basis for clinical decision-making and advancing the development of neoadjuvant immunotherapy, ultimately improving patient prognosis and quality of life.

### Importance of personalized treatment

Although the development of NIT is promising, only some patients benefit, making personalized treatment crucial. This approach enhances treatment efficacy by matching plans and adjusting dosages based on the patient’s tumor biological characteristics and individual tolerance. It also reduces adverse reactions by assessing individual factors to predict and mitigate the risk of immune-related side effects, thereby avoiding drug toxicity. Furthermore, personalized treatment improves prognosis by tailoring plans to each patient’s specific circumstances, aiming to increase the pathologic response rate, extend survival, and focus on quality of life. Subsequent treatment strategies should be individualized based on the patient’s disease status, tumor molecular characteristics, PD-L1 expression, and overall health. The use of biomarker testing to screen for compatible strategies can increase treatment efficacy and safety.

### Limitations

This study has several limitations, similar to previous bibliometric analyses. First, the selection of the Web of Science, PubMed, and Scopus databases means that the citation counts for some studies could not be fully assessed. Second, the literature search included article published until only September 10, 2024, and it did not comprehensively include publications from 2024. Future research should consider adopting a more comprehensive approach incorporating diverse data sources and languages. Nonetheless, the analysis in this study still provides valuable insights into the field’s current state.

### Conclusion and future outlook

In summary, NIT demonstrates significant potential in the treatment of NSCLC. Immunotherapy can effectively control tumor progression and improve long-term survival rates by activating the patient’s immune system. The application of radiomics provides crucial support for personalized treatment, helping clinicians better understand tumor biological characteristics, predict treatment responses, and monitor outcomes. Future research should continue to explore the mechanisms of immunotherapy, optimize clinical trial designs, and investigate personalized treatment strategies to enhance treatment efficacy and quality of life for NSCLC patients. As our understanding of immunotherapy deepens and new technologies and techniques are explored, NIT is expected to become a standard for treating NSCLC.

## Data Availability

The original contributions presented in the study are included in the article/supplementary material. Further inquiries can be directed to the corresponding author.
